# Disrupting the rhythm of depression using Mobile Cognitive Therapy for recurrent depression: randomized controlled trial design and protocol

**DOI:** 10.1186/1471-244X-11-12

**Published:** 2011-01-14

**Authors:** Claudi LH Bockting, Gemma D Kok, Lillian van der Kamp, Filip Smit, Evelien van Valen, Robert Schoevers, Harm van Marwijk, Pim Cuijpers, Heleen Riper, Jack Dekker, Aaron T Beck

**Affiliations:** 1 Department of Clinical and Experimental Psychology, Groningen University, Groningen, The Netherlands; 2 Centre of Prevention and Early Intervention, Trimbos Institute (Netherlands Institute of Mental health and Addiction), Utrecht, The Netherlands; 3 Department of Epidemiology and Biostatistics, VU University medical centre, Amsterdam, The Netherlands; 4 Netherlands Center for Occupational Diseases, Coronel Institute of Occupational Health, Academic Medical Center, University of Amsterdam, Amsterdam, The Netherlands; 5 Department of Psychiatry, University Medical Center Groningen, Groningen, The Netherlands; 6 Department of General Practice and the EMGO Institute for Health and Care Research (EMGO+), VU University medical centre, Amsterdam, The Netherlands; 7 Department of Clinical Psychology of the Vrije Universiteit, Amsterdam, The Netherlands; 8 Mental Health Care Center Arkin/PuntP, Amsterdam, The Netherlands; 9 Department of Psychiatry, University of Pennsylvania, Philadelphia, USA

## Abstract

**Background:**

Major depressive disorder (MDD) is projected to rank second on a list of 15 major diseases in terms of burden in 2030. The major contribution of MDD to disability and health care costs is largely due to its highly recurrent nature. Accordingly, efforts to reduce the disabling effects of this chronic condition should shift to preventing recurrence, especially in patients at high risk of recurrence. Given its high prevalence and the fact that interventions are necessary during the remitted phase, new approaches are needed to prevent relapse in depression.

**Methods/design:**

The best established effective and available psychological intervention is cognitive therapy. However, it is costly and not available for most patients. Therefore, we will compare the effectiveness and cost-effectiveness of self-management supported by online CT accompanied by SMS based tele-monitoring of depressive symptomatology, i.e. Mobile Cognitive Therapy (M-CT) versus treatment as us usual (TAU). Remitted patients (n = 268) with at least two previous depressive episodes will be recruited and randomized over (1) M-CT in addition to TAU versus (2) TAU alone, with follow-ups at 3, 12, and 24 months. Randomization will be stratified for number of previous episodes and type of treatment as usual. Primary outcome is time until relapse/recurrence over 24 months using DSM-IV-TR criteria as assessed by the Structured Clinical Interview for DSM-IV Axis I Disorders (SCID). For the economic evaluation the balance between costs and health outcomes will be compared across strategies using a societal perspective.

**Discussion:**

Internet-based interventions might be helpful in empowering patients to become their own disease managers in this lifelong recurrent disorder. This is, as far as we are aware of, the first study that examines the (cost) effectiveness of an E-mental health program using SMS monitoring of symptoms with therapist support to prevent relapse in remitted recurrently depressed patients.

**Trial registration:**

Netherlands Trial Register (NTR): NTR2503

## Background

Major depressive disorder (MDD) is projected to rank second on a list of 15 major diseases in terms of burden in 2030 [1]. The contribution of MDD to disability and health care costs is largely due to its highly recurrent nature [2,3]. Accordingly, efforts to reduce the disabling effects of depression should shift to preventing recurrence, especially in patients at high risk of recurrence. Current maintenance therapy is often labour intensive involving collaboration among multiple health services over long periods. This is costly and prone to non-adherence to protocols on the part of health service providers and non-compliance on the part of patients. In this context it is essential to empower patients to become their own disease managers.

Cognitive therapy (CT) is an effective treatment of MDD and an effective preventive treatment [4-6]. In a multicenter RCT enrolling remitted recurrently depressed patients, we evaluated the efficacy and cost-effectiveness of a brief face-to-face CT added to treatment as usual (TAU) compared with TAU alone [6]. In line with other studies on CT, we found that CT was effective (and cost-effective: Bockting CLH, Dijkgraaf MGW, Hakaart-van Roijen L et al.: Cost-effectiveness of relapse-prevention cognitive therapy in recurrent depression: a two year study, submitted) in preventing recurrences over a 2-year follow-up and even over 5.5 years [7], in patients with multiple previous episodes.

Given its high prevalence and the fact that interventions are necessary during the remitted phase of this life-long disease, new approaches are needed to prevent relapse and recurrence in depression. This new approach must not only be acceptable to remitted patients, but also reach patients who often do not seek treatment in this phase of the disease. Several advantages have been noted of an e-mental health disease management program [8,9]. First, SMS-based monitoring on depression makes it easier for the patient and therapist to detect relapse as early as possible. Second, internet-based delivered cognitive therapy including SMS based monitoring by making use of cell phones (Mobile-CT) is mainly a self management intervention in which patients create their own prevention of relapse program. Third, patients can easily dose their own amount of online therapist support in line with their needs. Overall, therapist's involvement may be reduced, as has been reported in the internet based treatment of acute depression [8]. Finally, this self-management approach toward preventing relapse and recurrence in depression can be used at home or at any venue of convenience to the patient. A recent meta-analysis [9] revealed that internet based interventions seem to be effective interventions for acute depression, especially when the intervention is supported by therapist contact.

## Trial objectives and Purpose

In this study, the addition of an internet based intervention with automated tele-monitoring (Mobile-CT, referred to as M-CT) to TAU, will be compared to TAU alone in a sample size of 214 (2x107) recovered patients. Alongside the randomized controlled trial, a cost effectiveness analysis (from a societal perspective) will be conducted.

It is hypothesized that adding M-CT is clinically superior to treatment-as-usual alone (TAU) for preventing relapse and recurrence in depressive disorder. In addition, we expect that the intervention dominates the comparator condition in terms of cost-effectiveness. Since co-morbidity with concurrent chronic somatic illnesses, is defined by the American Psychiatric Association [10] as a risk factor for future relapse and recurrence, differential response in this group of patients will be examined explicitly. We will also conduct moderator analysis to see if there are any baseline characteristics of the participants that are prognostically relevant for treatment response. Finally, we will conduct incremental cost-benefit regression analysis to identify subgroups where the intervention is particularly cost-effective.

## Methods/Design

In this randomized controlled trial with a sample size of 268 participants (after accounting for 20% drop out, M-CT: 107, TAU: 107) we compare an internet-supported self-directed prevention of relapse program as part of a SMS based monitoring versus treatment as usual (TAU). This M-CT program is called *Depression Free*. The target population is a group at elevated risk of relapse and recurrence as identified in several guidelines (e.g. NICE) [11,12] that consumes a considerable amount of health care and for whom initial benefits of antidepressants (AD) may be wane off in the long run. Relapse rates rise with increasing numbers of previous episodes up to 70% in 5 years [12]. In our previous study, we observed up to 62% recurrences within 2 years [13].

**Figure 1:**
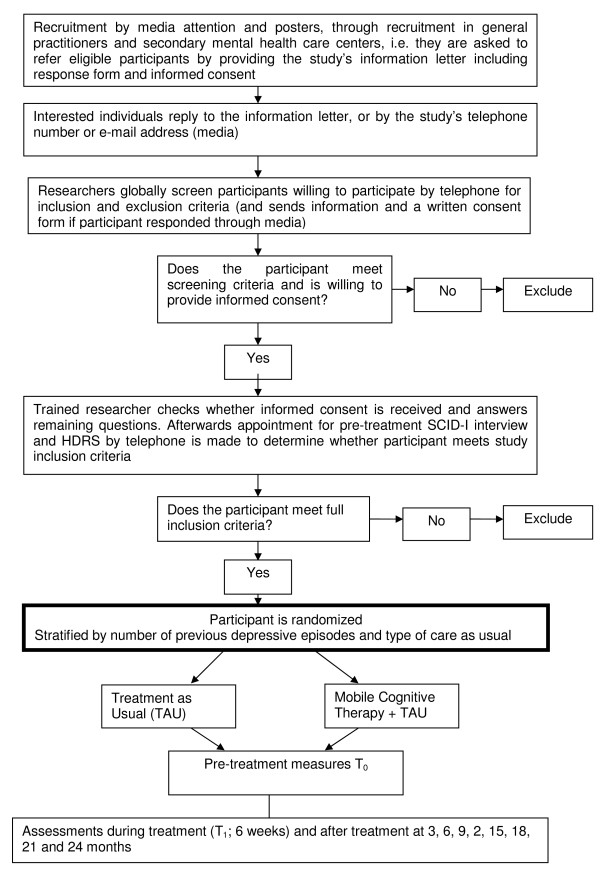
**Flow Diagram**.

Randomization will be undertaken by an independent researcher and will be stratified by the number of previous depressive episodes and type of care (i.e. care by a general practitioner versus care in a mental health center) will then be used for stratified randomization. Thereafter our researchers receive the participant number and the automatically random generated condition in the trial by email. For a Flow diagram of the assessment methods see Figure 1.

We monitor the primary outcome (relapse) over a period of 24 months. Assessments by trained assessors who are blind to treatment allocation (and whose blindness is checked within each assessment session) take place directly after the start of the treatment at three months, 12 and 24 months. For the research aims focused on potential working mechanisms of M-CT we added in between self-report assessments, i.e. baseline, 1.5 months and 3 months.

### The interventions

*M-CT-arm*: This M-CT treatment builds on a previously evaluated face to face intervention, i.e. Preventive Cognitive Therapy (PCT) [14] and has been developed in collaboration Bockting & van Valen [15] with the Trimbos Institute. The face to face PCT is an adapted type of cognitive therapy for acute depression [16] and specifically developed to prevent relapse in recurrent depression in remitted patients. It consists of eight sessions. Like in regular CT, each PCT session follows a fixed structure, with agenda setting, review of homework, explanation of rationale of each session, and assignment of homework. A manual describing the structure of the treatment and interventions used is available [14]. The intervention prevention program targets underlying cognitive vulnerability factors, such as depressogenic assumptions. Unlike CT for acutely depressed patients, PCT is not primarily directed toward modifying negative thoughts. Instead, it starts with the identification of negative thoughts and dysfunctional attitudes, aided by a self-report questionnaire with examples of attitudes and specific techniques such as the downward arrow technique. The focus of treatment is then directed on changing these attitudes using different cognitive techniques such as Socratic questioning and identification of positive attitudes. Moreover, patients are encouraged to practice with alternative attitudes in the final sessions. Remitted patients with a history of recurrences have an inability to retrieve specific memories from the past and this is associated with impaired problem-solving skills [e.g. 17], long-term course of depressive disorders [18] and difficulties in recovering from depression. Part of the treatment is keeping a diary of positive experiences in order to enhance specific memories of positive experiences, instead of retaining overly general memories. Further specific relapse/recurrence prevention strategies are formulated in the last three sections of the M-CT resulting in a personal prevention plan.

The M-CT intervention is based on the above described face to face PCT. It is offered over the internet in a series of 8 well-structured modules with online therapist contact (with a maximum of 4 telephone sessions) and by SMS. Each module includes assignments with automatically generated feedback. In addition, films can be activated by the participant to explain more about specific topics. Each module can be completed in approximately 20 minutes, not counting the time required to complete additional assignments. If all assignments are completed, E-personal prevention book will be automatically generated that can be of help in case of relapse of lowered mood. Automated checks will be conducted to ascertain that participants have not only completed modules, but also understood them correctly, before they can move on to the next. When participants do not log in on the intervention's website they will receive friendly reminders by SMS or mail to proceed with the intervention.

Depression-related outcomes (changes in mood) will be monitored with help of SMS (or by email if requested). To this end, participants periodically receive SMS messages in which they will be asked to rate their mood. Participants have to answer by sending a message back consisting of a number with which they rate their mood. When depressed mood is present and appears to persist, then the frequency of messages is increased and other depressive symptoms are monitored also using a web-based self-report assessment (IDS-R) [19]. In case there is indeed an indication for a depressive episode, participants will be interviewed using the SCID [20] and the Hamilton Rating Scale for Depression (HRSD) [21]. This allows for the early detection of possible depression onset. In that scenario, participants receive advice and are encouraged to return to the website where they can find 'prescribed' modules. Hopefully, this offers them the opportunity to better cope with lowered moods.

The intervention is designed to be easily accessible, acceptable and as non-intrusive as possible, while at the same time allowing for tele-monitoring of health related outcomes over the time-span of several years. The web-based intervention has been developed by the Trimbos Institute, the University of Groningen, CrossOver Consultancy and their partners.

#### Treatment As Usual

The intervention will be compared to treatment as usual (TAU). In this context TAU is fairly heterogeneous: it typically consists of antidepressant (AD) maintenance medication in primary and secondary care, counseling or face-to-face PCT in secondary care, but often there is no treatment at all. To compare the intervention with TAU is relevant from a public health perspective: it would help to demonstrate the intervention's added value over and above TAU. We will not intervene with TAU, but monitor TAU using a health service receipt interview, the TIC-P [22]. We will assess compliance and adherence to AD use, but also the use of the M-CT program.

### Sample size

With 107 in M-CT versus in TAU 107 participants per condition the trial will be powered to detect a difference of 20% in the cumulative incidence rate of relapse/recurrence with a 2 year follow-up in a 2-tailed test at the conventional alpha level of 0.05 and a power of (1-beta) = 0.80, while conservatively assuming that relapse/recurrence will occur in 50% of the cases. Allowing for a drop-out of 20% we need to include 268 participants at baseline.

### Referral and recruitment

Patients will be recruited by media (announcements, banners placed in various related websites, media attention in interviews), referral by general practitioners and mental health services. Patients with concurrent chronic somatic illnesses will be recruited by targeted marketing strategies (e.g. banners on website targeted on patients with chronic somatic illnesses, posters in hospitals) and specific recruitment at general practitioners.

### Inclusion criteria

We include recovered patients with a history of at least two previous depressive episodes in the past five years. The last episode has to be at least 2 months and no longer than 2 years ago. The last episode has to be at least 2 months and no longer than 2 years ago and a current score of  ≤10 on the HRSD [21; in line with other prevention studies, e.g. 5-6]. No restriction with respect to co-morbidity on Axis II and III, i.e. a concurrent chronic somatic illness is defined as risk factor for relapse and recurrence, [APA, 10]. Consenting participants need to be fluent in Dutch and have access to the internet.

### Exclusion criteria

Exclusion criteria are: current mania or hypomania or a history of bipolar illness, any psychotic disorder (current and previous), alcohol or drug misuse, predominant anxiety disorder.

### Assessment of Eligibility and Baseline Measures

#### Informed consent

We inform patients about the study before they come into the study in two ways. First, by informing the patient through a therapist or a general practitioner (GP). If a therapist/GP wants to inform the patient himself, the patient then receives the information via the therapist/GP and is given a letter containing all the information. If the patient is interested in participating, then the participant will contact the researcher. Subsequently, the researcher checks that the participant understands all aspects of the trial. If the participant agrees to enter the trial, she completes a copy of the consent form and sends it to the researcher.

The second procedure we use is by directly informing the patient. Participants than initiate that contact with the researcher themselves (mostly informed by media/websites or by their former therapist/GP with a letter, by advertisements or interviews). Subsequently, the researcher informs the participant, the participant receives the information in a letter with all the information in it. If the participant is still interested in participating then the researcher checks that the participant understands all aspects of the trial. If they agree to enter the trial, they complete a copy of the consent form and send it to the researcher. We remind participants that they can withdraw from the trial at any time and that this has no consequences for their treatment as usual.

We ask consenting participants to provide information about their socio-demographic background and assess their eligibility in more detail using semi-structured clinical interviews (SCID-I, by telephone) and self-completed questionnaires (web-based). The researchers assess current and past diagnostic status using the Structured Clinical Interview for DSM IV (SCID) [20] and the Hamilton Rating Scale for Depression (HRSD) [21]. They ask participants to describe past and current treatments for depression and use of antidepressants. If participants meet all inclusion and none of the exclusion criteria for the study, they enter the study.

### Withdrawal

Participants can withdraw from treatment or from the study at any time.

### Safety monitoring and reporting

The trial protocol has been approved by an independent medical ethics committee (METIGG). Eligible people will only be included as participants in the trial after informed consent has been obtained.

We record and report suspected serious adverse events to the Multi-center Ethic Committee (METIGG) according to their individual guidelines.

#### Baseline assessment

For the baseline assessment we ask participants themselves to complete the web-based self-report questionnaires in two packages, i.e. explicit and implicit measures. The first part with assessments starting directly within a week, the second part containing implicit measures will be offered within 2 days after completion of self-report assessments measures. The following self-report measures will be used: the Inventory of Depressive Symptomatology, IDS-SR [19], Nemesis Somatic illnesses list [23], negative life events (Life events questionnaire, LGV) [24], self-esteem (Self-esteem Questionnaire) [25], personality pathology (Personality Diagnostic Questionnaire, PDQ-4) [26], everyday problems (EPCL) [27], hypomania (HCL-32) [28], direct and indirect costs (TIC-P) [22] and Medication Adherence Questionnaire (MAQ) [29], a measure of general quality of life (Euro-QOL EQ-5D) [30], and rumination (Ruminative Responses Subscale of the Response Styles Questionnaire RSQ) [31], dysfunctional attitudes (Dysfunctional Attitudes Scale, DAS) [32], LEIDS [33], acceptance (Acceptance and Action Questionnaire, AAQ) [34], coping (Utrecht Coping List, UCL) [35], Mastery 7 [36]. After 6 weeks this set will be repeated with the exception of the TIC-P, LGV, PDQ, MAQ EQ-5 D and Nemesis Somatic illnesses list. During follow-up every three months the following self-report assessments will be repeated: IDS-SR, HCL-32, TIC-P, EPCL, Mastery7 and EQ-5 D will be administered. For a complete overview of the assessments see Table 1. Participants in the M-CT group will also answer questions of the Dutch version of the Credibility and expectancy questionnaire [37,38] before and after finishing M-CT.

**Table 1:** Overview of assessments

Measure	Description	T_0_	T_1_	T_2_	T_3_	T_4_	T_5_	T_6_	T_7_	T_8_	T_9_*
*Interviews*											

SCID-I	DSM-IV-TR Axis I disorders	+		+			+				+

HDRS	Depressive symptoms and severity	+		+			+				+

CEQ, only M-CT group	Credibility/expectancy questionnaire	+		+							

*Implicit computer assignments*											

IAT	Implicit associations	+		+							

RSVP	Ability to disengage from negative information	+		+							

*Self report measures*											

IDS-SR	Depressive symptoms	+	+	+	+	+	+	+	+	+	+

RSQ	Ruminative responses	+	+	+			+				+

EQ-5D	Quality of life	+		+	+	+	+	+	+	+	+

DAS	Dysfunctional Attitudes	+	+	+			+				+

AAQ	Experiential acceptance and avoidance	+	+	+			+				+

UCL	Coping	+	+	+			+				+

LGV	Life-events	+		+			+				+

Self-esteem	Self-esteem										

PDQ-4+	Personality	+					+				+

Nemesis Somatic illnesses list	List of somatic disorders	+					+				+

EPCL	Everyday problem list	+	+	+	+	+	+	+	+	+	+

HCL-32	Hypomania	+	+	+	+	+	+	+	+	+	+

TIC-P including MAQ**	Direct/indirect costs	*+*		+	+	+	+	+	+	+	+

LEIDS	Dysfunctional attitudes	+	+	+			+				+

Mastery 7	Mastery	+	+	+	+	+	+	+	+	+	+

*T0 = Baseline, T1 = +1,5 month, T2 = 3 months, *T3 = 6 months, T4 = 9 months, T5 = 12 months, T6 = 15 months, T7 = 18 months, T8 = 21 months, T9 = 24 months*

**MAQ: Medication Adherence questionnaire

### Outcome measures

For an overview of the assessments at baseline, in between- and post treatment and follow up assessments see Table 1.

#### Primary outcome

Primary outcome is time until relapse or recurrence of depression in the experimental group relative to the control group over 24 months using DSM-IV-R criteria as assessed by the SCID at 3 months, 12 months and 24 months [20]. Co-morbidity with concurrent chronic somatic illnesses will be assessed using the NEMISIS somatic illnesses list [23]. Secondary outcome is symptom severity as measured with the Inventory of Depressive Symptomatology (IDS-R) [19] and number of relapses as assessed by the SCID [20]. For the economic evaluation we will use the EuroQoL (EQ-5D) to obtain a generic quality of life related outcome [30]. Cost data related to health care uptake will be sampled using the TIC-P [22]. Cost data stemming from production losses due to absenteeism and working less efficiently while at work will be collected with the specific modules from the TIC-P [22].

#### Moderators and Mediators

For potential moderators (illness related, stress-related and cognitive-related) predictors and mediators the following self-report measures will be used at baseline, at 1,5 and at 3 months (internet based): Inventory of Depression Symptomatology (IDS-SR every 3 months) [19], Dysfunctional Attitude Scale, (DAS-A) [32], LEIDS [33], Everyday Problem Checklist (EPCL) [27], Negative Life Events Questionnaire [24] and to assess nonadherence to AD with the Medication Adherence Questionnaire (MAQ) [29], Mastery7 [36]. To enable calculating quality adjusted life years required for the economic evaluation the EQ5 D will be administered every 3 months [30]. To test whether M-CT affects implicit attitudes and attentional bias differentially and whether residual difficulty to disengage and residual dysfunctional implicit attitudes are related to the return of depressive symptoms, an web-based Implicit Association Test (IAT) [39] will be used to assess implicit attitudes. A web-based rapid serial visual presentation (RSVP) [40] task will be used to assess the difficulty to disengage from negative information. Difficulty to disengage will be indexed by the magnitude of the attentional blink when negative self descriptors are presented as the first target and neutral words as the second. For an overview of the assessments at baseline, in between- and post treatment and follow up assessments see Table 1.

### Analysis

Cox regression analysis (survival analysis) will be performed, including as covariates the stratification variables that will be used in randomization, i.e.: number of previous episodes and type of care (primary/secondary/no care). Analysis will be conducted by intention to treat, including all subjects randomized in the study, and per protocol, including only subjects satisfying protocol. Statistical significance will be set at *P *< .05.

Mixed-model analysis of variance (ANOVA) will be used for the other variables, including baseline values of the dependent variable as a covariate in all analyses. We shall use implicit and explicit cognitive measures and stress measures (daily hassles) to explore the extent to which they mediate relapse and recurrence during treatment and follow up.

For the economic evaluation the balance between costs and health outcomes will be compared across strategies using a societal perspective. Primary outcome measure: the number of depression-free days. Both short-term and long-term consequences will be compared. Additionally, Quality Adjusted Life Years will be used as outcome (see also Table 1).

## Discussion

Given the high prevalence of MDD and its recurrent character new minimal interventions are needed to prevent relapse and recurrence in depression. Internet-based interventions might be helpful in empowering patients to become their own disease managers in this lifelong recurrent disorder. This is, as far as we are aware of, the first study that examines the (cost) effectiveness of an E mental health program using SMS and mail monitoring of symptoms with therapist support to prevent relapse in remitted recurrently depressed patients. Attrition is a very common phenomenon in internet-based interventions, hopefully the therapist support in this intervention will reduce attrition rates, as suggested in the meta-analysis of Spek et al [9]. In addition, mediation variables will be examined to get more insight into the most effective ingredients of the M-CT used. This might lead to insights that will lead to the development of more targeted interventions.

In summary, given the highly recurrent nature of MDD, new minimal interventions should be developed and evaluated to prevent recurrence in patients at high risk of recurrence, i.e. patients with multiple prior episodes. Internet based intervention including SMS based monitoring might be promising in disrupting the rhythm of depression, as will be examined in this study. This combination of self management-monitoring and self help could be an easily implemented and potential cost effective part of a broader disease-management program of a chronic (recurrent) illness, i.e. MDD.

## Competing interests

CB participated in a discussion on treatment for depression for a web-based course of Wyeth once on 1/11/2007.

All other authors declare that they have no competing interests.

## Authors' contributions

CB, GK drafted this paper which was added to and modified by all other authors. CB and EvV and GK modified the content of PCT to the content of an internet based Mobile CT, and LvdK and FS developed the technical part of de internet based CT. CB, EvV, FS, HvM, RS, PC, HR, JD and AB, contributed to the design of the study and CB and FS to the analytic strategy. All authors read and approved the final manuscript.

## Appendix 1: Statistical Analysis Plan

All analysis will be conducted in agreement with the intention to treat principle as per the CONSORT statement. Cox regression analysis will be performed, including as covariates the stratification variables that will be used in randomization, i.e.: number of previous episodes, type of care (no/primary/secondary). Statistical significance will be set at P < .05. When adding the intervention to TAU is superior then the relapse/recurrence rate in this condition should be smaller than in the comparator condition (TAU alone). Therefore, we will obtain cumulative relapse/recurrence hazard rate ratios (Hr's). To gauge the robustness of the outcomes, the above analyses will be repeated under a completers-only framework.

Since co-morbidity with a concurrent chronic somatic illness for which medical attention is received is defined by the American Psychiatric Association as risk factor for future relapse and recurrence [10], differential response in this group of patients will be examined explicitly. Subgroups that show particularly good response to the intervention will be identified by regressing SCID depression severity on the interaction term of treatment and clinical characteristics of the participants as measured at baseline. Examples of other characteristics are number of previous depressive episodes, age at which the first depression occurred, concurrent personality disorders, concurrent anxiety disorder, experienced life events, some demographic characteristics (like gender) and sense of mastery. In addition, moderator analysis will also be conducted for demographic variables such as gender, age, educational level, partner status, employment status. These variables have been shown to be of prognostic value in depressive disorder. The same set of predictor variables will also be used in an incremental net benefit regression analysis to addresses the research question in what groups the intervention is likely to be particularly cost-effective.

The economic evaluation will be conducted both as a cost-effectiveness analysis with depression-free survival time as the clinical end term, and as a cost-utility analysis with incremental costs per quality adjusted life years (QALYs) gained as the clinical endpoint. For the latter, health-related quality of life, will be assessed with help of the EQ-5 D at baseline and follow-ups. Direct medical and direct non-medical cost data are collected with the TIC-P [22], a widely used health service receipt interview in economic evaluations. Unit resource use (GP visits, hospital days, etc.) will be multiplied by their appropriate integral cost prices [41]. Indirect non-medical cost data related to production losses through work loss days and work cutback days will be sampled with specific modules of TIC-P [22]. For the economic evaluation use will be made of the pertinent guidelines [42-44]. In other words, analyses will be conducted in agreement with the intention-to-treat principle, the societal perspective will be taken encompassing intervention costs, direct medical costs, direct non-medical costs and indirect costs. The latter will encompass production losses due to absenteeism and due to working less efficient while at work. Production losses will be economically valuated using the friction cost method [45] as per the Dutch guideline [42]. The time horizon will be set at two years and therefore costs and effects will be discounted. Costs and effects will be analyzed simultaneously, incremental cost-effectiveness ratios (ICERs) will be calculated and placed within 95% confidence intervals, 2,500 bootstrap replications of the ICERs will be projected on a cost-effectiveness plane, ICER acceptability curves will be plotted against different willingness-to-pay ceilings and sensitivity analysis will be conducted as a matter of course focusing on uncertainty in the analysis.

## Pre-publication history

The pre-publication history for this paper can be accessed here:

http://www.biomedcentral.com/1471-244X/11/12/prepub
